# High prevalence of anti-SARS-CoV-2 IgG antibody in the Xikrin of Bacajá (Kayapó) indigenous population in the brazilian Amazon

**DOI:** 10.1186/s12939-021-01392-8

**Published:** 2021-01-28

**Authors:** Eliene Putira Sacuema Rodrigues, Isabella Nogueira Abreu, Carlos Neandro Cordeiro Lima, Dennyson Leandro Mathias da Fonseca, Sávio Felipe Gomes Pereira, Laena Costa dos Reis, Izaura Maria Vieira Cayres Vallinoto, João Farias Guerreiro, Antonio Carlos Rosário Vallinoto

**Affiliations:** 1 Laboratório de Genética Humana e Médica, Instituto de Ciências Biológicas da Universidade Federal do Pará, Belém, PA Brasil; 2 Laboratório de Virologia, Instituto de Ciências Biológicas da Universidade Federal do Pará, Belém, PA Brasil; 3 Universidade da Amazônia, Belém, Pará Brasil; 4 Programa de Pós-graduação em Enfermagem, Instituto de Ciências da Saúde da Universidade Federal do Pará, Belém, PA Brasil

**Keywords:** COVID-19, Xikrin, Indigenous peoples, Amazonia

## Abstract

The COVID-19 pandemic caused by Severe Acute Respiratory Syndrome Coronavirus 2 (SARS-CoV-2) reached the Brazilian Amazon and spread among indigenous populations. In the present study, we demonstrate a high prevalence of infection among the Xikrin of Bacajá people (Kayapó). A sample of 100 individuals of both sexes (51 men and 49 women) with ages ranging from 2 to 82 years were clinically evaluated and tested for the presence of anti-SARS-CoV-2 IgG antibody. Among all investigated individuals, 58 were IgG-reactive (58 %) by a rapid test, and 73 (73 %) were reactive in an enzyme-linked immunosorbent assay, with no difference between sexes. Oxygen saturation ranged from 82 to 99 %, with the lowest value observed in a two-year-old girl. The results show that as expected, SARS-CoV-2 infection rapidly reached more than 70 % of the population, most likely because of the difficulties of maintaining social distance due to cultural characteristics. These results highlight the importance of indigenous health policies as a means of minimizing the impact of the pandemic on these communities.

## Background

After the identification of the new Severe Acute Respiratory Syndrome Coronavirus 2 (SARS-CoV-2) and the first cases of Coronavirus Disease 2019 (COVID-19) in Wuhan, China, in November 2019, the World Health Organization declared a pandemic alert to all nations [1], increasing concern among health authorities regarding the possible impact of the pandemic if it reached vulnerable populations, such as the indigenous peoples of the Brazilian Amazon [2, 3].

Vulnerability to new infectious agents has been attributed to indigenous peoples because of the characteristics of their genetically controlled immunological response to infections [4, 5]. In addition, we do not know whether these groups may be more vulnerable to SARS-CoV-2 infection in the presence of other coinfections and pre-existing conditions, such as obesity and malnutrition [4, 6].

Recently, our group published a warning about the importance of conducting seroepidemiological surveillance studies in indigenous, riparian, and quilombola communities of the Brazilian Amazon [4] because these populations are characterized by distinct cultural and health aspects that may affect the spread dynamics of SARS-CoV-2. Thus, we started a seroepidemiological surveillance study in indigenous communities of the state of Pará (Northern Brazil) to evaluate the seroprevalence of anti-SARS-CoV-2 antibodies in these communities and the impact of the virus on the collective health of these peoples.

## COVID-19 in the xikrin of Bacajá village

**Fig. 1:**
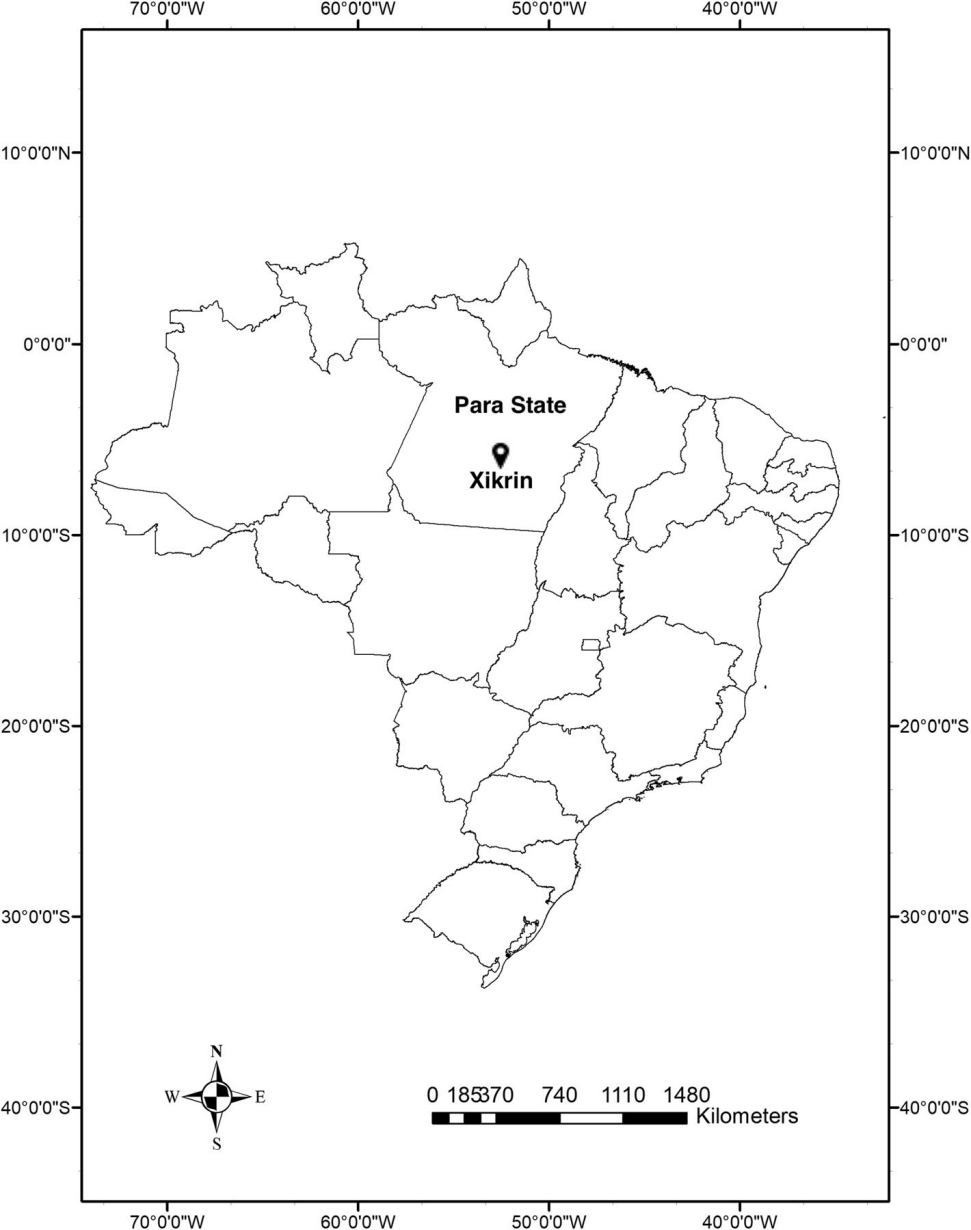
Map of Brazil showing the geographical location of the Xikrin of Bacajá reserve. An area of 1,650,000 ha located between the municipalities of São Félix do Xingú and Senador José Porfírio, state of Pará, Northern Brazil, i.e., the location of the Xikrin (Kayapó) population

With the help of a multidisciplinary health team from the Health Department of the State of Pará (Secretaria de Saúde do Estado do Pará – SESPA) and the Special Indigenous Health District of Altamira (Distrito Sanitário Especial Indígena de Altamira – DSEI-Altamira) of the Special Secretariat of Indigenous Health (Secretaria Especial de Saúde Indígena – SESAI-MS), we evaluated the prevalence of anti-SARS-CoV-2 IgG antibodies using a rapid test (lateral flow method; Guangzhou Wondfo Biotech Co., China) and an enzyme-linked immunosorbent assay (ELISA; Anti-SARS-CoV-2 S1 IgG, Euroimmun, Brazil) in the Xikrin of Bacajá indigenous population (Kayapó), Mebengokré linguistic group of the Macro-Jê linguistic trunk, located on the left margin of the middle Bacajá River (Fig. 1), a tributary of the right margin of the Xingu River, between the municipalities of São Félix do Xingú and Senador José Porfírio, Pará state, Brazil. The present study was approved by the leaders of the Xikrin people and by the National Research Ethics Committee (Comissão Nacional de Ética em Pesquisa - CONEP) (CAAE: 33470020.0.1001.0018). The visit to the community occurred in September, when the population was experiencing active cases of COVID-19.

A sample of 100 individuals from the Kenkrô, Bacajá, Pykatum, Moinorô, Mrotidjam, and Pytako villages of both sexes (51 men and 49 women) with ages ranging from 2 to 82 years (mean 27.9) were clinically evaluated and tested for the presence of anti-SARS-CoV-2 IgG antibody. A total of 58 individuals were IgG-reactive (58 %) by the rapid test, and 73 (73 %) were reactive according to the ELISA, with no significant difference between sexes (men [54.2 %] and women [45.8 %]; *p* = 0.5672). The seroprevalence rates among the villages according to the ELISA were as follows: Pykatum (0 %; 0/4), Bacajá (30.4 %; 7/23), Kenkrô (90.2 %; 37/41), Mrotidjam villages (100 %; 1/1), Moinorô (100 %; 13/13), and Pytako (100 %; 19/19). The mean age of seropositive individuals was 23.9 years, and the mean age of seronegative individuals was 40.5 years. Oxygen saturation ranged from 82 to 99 % (mean 97.8 %), and the lowest value was observed in a two-year-old girl. The heart rate ranged from 54 to 127 bpm (mean 80.3 bpm).

Most individuals who were seropositive for IgG (79.5 %) reported no symptoms associated with COVID-19. Among the 15 individuals who were symptomatic (20.5 %), the main symptoms reported were cough, dyspnea, fatigue, diarrhea, ear pain, headache, and chest and back pain. Only one death, a 34-year-old man serving as chief (*cacique*) of the Xikrin people, was recorded among the seropositive individuals within the sample (1.37 % case fatality rate) during our visit to the Xikrin village.

The body mass index (BMI) of the population ranged from 13.3 to 18.3 kg/m^2^ in children aged 0 to 10 years (mean = 16.6 kg/m^2^); from 18.9 to 29.1 kg/m^2^ in young adults (mean = 23.2 kg/m^2^), and from 20.3 to 34.5 kg/m^2^ in adults (mean = 25.7 kg/m^2^). The only case of malnutrition occurred in a five-year-old child (13.3 kg/m^2^) who was nonreactive for anti-SARS-CoV-2 IgG. Four cases of obesity were observed, including one child (four years old) seronegative for IgG and three adults reactive for IgG (one man [aged 50 years] and two women [aged 20 and 33 years]).

Regarding coinfection, human T-lymphotropic virus type 2 (HTLV-2) is hyperendemic in the Xikrin population, with prevalence rates above 30 % [7]. The presence of this retrovirus in the coinfection condition has been implicated as a modulating factor of other infections, such as those caused by the hepatitis B (HBV) and C (HCV) viruses [8]; however, in the case of SARS-CoV-2, no data indicate possible modulation of COVID-19 due to HTLV-2/SARS-CoV-2 coinfection.

In addition to the presence of coinfections, other social and environmental factors, such as a lack of drinking water and malnutrition, may impact the spread and severity of COVID-19 in indigenous populations [4]; notably, however, in the Xikrin population, only one case of malnutrition and four cases of obesity were observed, which may be factors associated with the higher number of asymptomatic and mild cases of COVID-19; only one death was recorded in the village.

The results show that as expected, SARS-CoV-2 infection quickly reached 73 % of the population, suggesting that the population would have achieved herd immunity [4, 9]. We speculate that this rate is most likely a consequence of the difficulties of maintaining social distance due to cultural characteristics, such as different families residing in the same *maloca* (indigenous home). Additionally, according to personal information provided by an SESAI health care staff member during the visit, some members of the community had traveled to neighboring villages with cases of COVID-19, allowing the virus to be introduced to the village after these individuals returned. The high prevalence observed in the Xikrin village was also reported in Manaus, the capital of Amazonas state, where 76 % of the population had detectable IgG antibodies in October [10]. On the other hand, the seroprevalence of anti-SARS-CoV-2 antibodies measured by rapid tests in 132 Brazilian cities in the months of May and June showed percentages that varied from 0 to 25.4 % [11], which highlights the heterogeneity of the pandemic in the Brazilian territory, reinforcing the speed at which the pandemic can reach indigenous populations in the Amazon due to their sociocultural and demographic characteristics, which expose these communities to rapid spread of the virus.

Notably, the present study used both rapid tests and ELISAs for antibody detection; based on the results of some studies, the rapid test has low sensitivity depending on the time of infection and may yield false-negative results as well as false-positive results due to cross-reaction [12, 13]. In addition, examination of the kinetics of the appearance of anti-SARS-CoV-2 antibodies has revealed the absence of seroconversion or even the loss of antibodies after infection [12, 14, 15]. Thus, we selected a method with greater sensitivity and specificity—ELISA—to more accurately detect the prevalence of anti-SARS-CoV-2 IgG antibody in the Xikrin people, which was found to be even higher according to this assay compared to the rapid test.

## Conclusions

In conclusion, the results presented here reinforce the importance of seroepidemiological surveillance studies, which we suggested previously [4], especially studies using methods with high sensitivity/specificity to minimize false-negative results. The results also highlight the importance of indigenous health policies as a means of monitoring and minimizing the impact of the pandemic on these communities, such as access to diagnosis, drinking water, hygiene materials, and emergency financial aid, through the creation of a national contingency plan for prevention and control of the epidemic in a more up-to-date manner and the establishment of a national vaccination plan that prioritizes indigenous peoples [4]. To the best of our knowledge, no reports are available in the scientific literature on COVID-19 in indigenous Brazilians. Thus, the primary purpose of the present study is to provide the Brazilian government with information on the pandemic among indigenous peoples to enable the development of adequate measures to control the epidemic in Brazilian indigenous communities, especially in the Amazon.

## Data Availability

Not applicable.

## Abbreviations

SARS-CoV-2: severe acute respiratory syndrome coronavirus 2
COVID-19: Coronavirus Disease 2019
SESPA: Health Department of the State of Pará (Secretaria de Saúde do Estado do Pará)
DSEI: Special Indigenous Health District (Distrito Sanitário Especial Indígena)
SESAI: Special Secretariat of Indigenous Health (Secretaria Especial de Saúde Indígena)
CONEP: National Research Ethics Committee
BMI: Body mass index
HCV: Hepatitis C virus
HBV: Hepatitis B virus
HTLV: Human T-lymphotropic virus
ELISA: Enzyme-linked immunosorbent assay
